# Additive Effect of Non-Alcoholic Fatty Liver Disease on Metabolic Syndrome-Related Endothelial Dysfunction in Hypertensive Patients

**DOI:** 10.3390/ijms17040456

**Published:** 2016-03-26

**Authors:** Maria Perticone, Antonio Cimellaro, Raffaele Maio, Benedetto Caroleo, Angela Sciacqua, Giorgio Sesti, Francesco Perticone

**Affiliations:** 1 Department of Experimental and Clinical Medicine, University Magna Græcia, Catanzaro 88100, Italy; mariaperticone@hotmail.com; 2 Department of Medical and Surgical Sciences, University Magna Græcia, Catanzaro 88100, Italy; antocime@hotmail.it (A.C.); sciacqua@unicz.it (A.S.); sesti@unicz.it (G.S.); 3 Unit of Cardiovascular Diseases, Azienda Ospedaliera Mater Domini, Catanzaro 88100, Italy; raf_maio@yahoo.it (R.M.); benedettocaroleo@libero.it (B.C.)

**Keywords:** endothelial dysfunction, non-alcoholic fatty liver disease, metabolic syndrome, cardiovascular disease and risk, arterial hypertension

## Abstract

Metabolic syndrome (MS) is characterized by an increased risk of incident diabetes and cardiovascular (CV) events, identifying insulin resistance (IR) and endothelial dysfunction as key elements. Moreover, non-alcoholic fatty liver disease (NAFLD) is bidirectionally linked with MS as a consequence of metabolic and inflammatory abnormalities. We addressed the question if the evolution in NAFLD might worsen endothelium-dependent vasodilating response in MS hypertensives. We recruited 272 Caucasian newly-diagnosed never-treated hypertensive outpatients divided into three groups according to the presence/absence of MS alone or in combination with NAFLD. MS and NAFLD were defined according to the National Cholesterol Education Program-Adult Treatment Panel III (NCEP-ATPIII) and non-invasive fatty liver index, respectively. We determined IR by using the homeostasis model assessment (HOMA) index. Vascular function, as forearm blood flow (FBF), was determined through strain-gauge plethysmography after intra-arterial infusion of acetylcholine (ACh) and sodium nitroprusside. MS+NAFLD+ group showed worse metabolic, inflammatory and vascular profiles compared with MS−NAFLD− and MS+NAFLD−. HOMA resulted in being the strongest predictor of FBF both in the MS+NAFLD− and in the MS+NAFLD+ groups, accounting for 20.5% and 33.2% of its variation, respectively. In conclusion, we demonstrated that MS+NAFLD+ hypertensives show a worse endothelium-dependent vasodilation compared with MS+NAFLD−, allowing for consideration of NAFLD as an early marker of endothelial dysfunction in hypertensives.

## 1. Introduction

Metabolic syndrome (MS) is a clinical condition characterized by a clustering of hemodynamic and metabolic risk factors including raised blood pressure (BP), atherogenic dyslipidemia, raised fasting glucose and central obesity [1]. All of these factors are interrelated and associated with an increased risk for incident diabetes and cardiovascular (CV) diseases [2,3]. Although the pathogenesis of MS remains not completely clarified, insulin resistance (IR) is believed to play a pivotal pathophysiological role in its development [4].

It is well recognized that endothelial dysfunction, primarily characterized by a reduced nitric oxide (NO) bioavailability, is an early step in the continuum of the atherosclerotic process. In addition, there are several lines of evidence demonstrating that it is a strong and independent predictor of CV events in different settings of patients [5,6], and that it is able to predict the appearance and progression of subclinical organ damage [6,7,8,9]. On the other hand, some experimental and clinical data have demonstrated that NO-mediated vasodilation is impaired in patients with IR [10,11,12], representing a possible pathogenetic mechanism linking MS to increased CV risk.

Non-alcoholic fatty liver disease (NAFLD) is bidirectionally linked with MS (13) as a consequence of the inflammatory and metabolic processes characterizing this condition. In keeping with this, previously published data demonstrated a strong relationship between IR and NAFLD [13,14,15,16]. It is plausible that, in visceral obesity, present in the MS, the excess of portal or intra-peritoneal fat promotes the appearance and progression of NAFLD by directly increasing the flux of free fatty acids to the liver [16]. Moreover, we recently reported that hypertensive patients with NAFLD show a significantly reduced endothelium-dependent vasodilation compared with hypertensives without NAFLD [17], confirming that the presence of more risk factors in the same setting of patients differentiates the risk profile of each subject.

However, at this moment, there are no data demonstrating if NAFLD has an additive effect in worsening endothelial function in subjects with MS. Thus, we designed the present study with the aim to demonstrate the additive effect of both MS and NAFLD on endothelium-dependent vasodilating response in hypertensive subjects.

## 2. Results

### 2.1. Study Population

Characteristics of the whole study population, stratified according to the presence/absence of MS alone or in combination with NAFLD, are reported in Table 1. In comparison with MS+NAFLD− patients, subjects in the MS+NAFLD+ group had significantly higher body mass index (BMI) and waist circumference. With regards to hemodynamic parameters, MS+NAFLD+ group showed higher systolic BP and pulse pressure (PP) values. As expected, MS+NAFLD+ patients exhibited higher gamma-glutamyltransferase (GGT), aspartate aminotransferase (AST) and alanine aminotransferase (ALT) values, and a worse metabolic and inflammatory profile, compared to the MS+LS− group.

### 2.2. Endothelium–Dependent and –Independent Vasodilation

The baseline forearm blood flow (FBF) did not differ among the three groups (Table 1). Intra-arterial infusion of achetylcholine (ACh) significantly increased FBF in a dose-dependent manner in all groups. The FBF values at the three incremental doses of ACh were 6.9 ± 3.0, 10.5 ± 4.6 and 16.3 ± 6.5 mL·100 mL^−1^ of tissue·min^−1^, 5.2 ± 2.2, 8.1 ± 3.9 and 12.7 ± 4.3 mL·100 mL^−1^ of tissue·min^−1^ and 4.8 ± 1.8, 6.9 ± 2.4 and 10.2 ± 3.7 mL·100 mL^−1^ of tissue·min^−1^ for MS−NAFLD−, MS+NAFLD− and MS+NAFLD+ groups, respectively.

**Figure 1:**
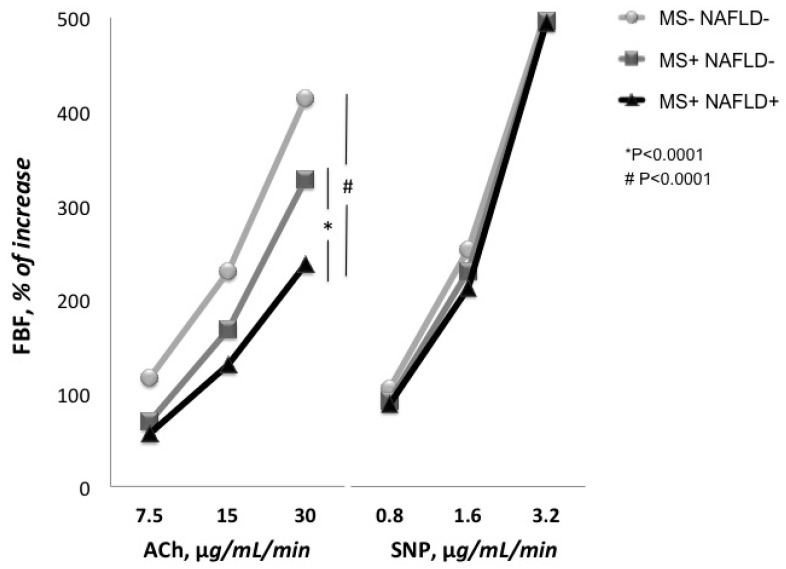
Responses of forearm blood flow (FBF) to intra-arterial infusions of acetylcholine (ACh) and sodium nitroprusside (SNP) in different groups.

As expected, the endothelium-dependent maximal vasodilating response to ACh was significantly (*p* < 0.0001) reduced in both MS+NAFLD− and MS+NAFLD+ groups in comparison with MS−NAFLD− group (Figure 1). In addition, MS+NAFLD+ patients showed a worse ACh peak percent increase when compared to the MS+NAFLD− group (Table 1). On the contrary, all patients showed a normal endothelium-independent vasodilation to sodium nitroprusside (SNP) infusions, without any significant difference among groups.

**Figure 2:**
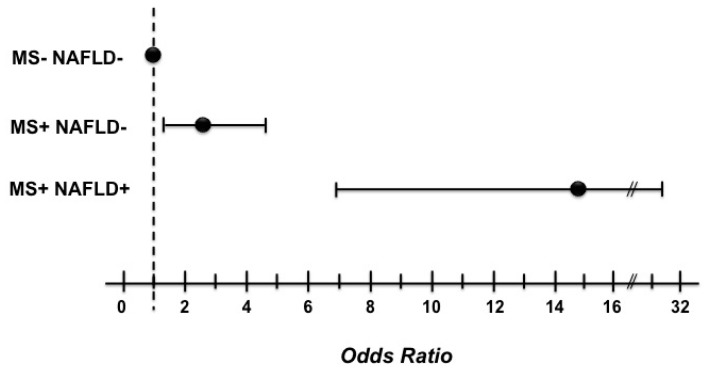
Graphic report of the logistic regression analysis for decreased forearm blood flow.

Finally, in the logistic regression model (Figure 2), patients with both MS and NAFLD had the highest risk for decreased FBF (OR = 14.81; 95% CI = 6.99–31.38; *p* < 0.0001), whereas the group with MS alone had an almost doubled risk (OR = 2.53; 95% CI = 1.32–4.86; *p* = 0.005).

### 2.3. Correlational Analysis

A linear regression analysis was performed to test the correlation between FBF and different covariates in the whole study population and in different groups (Table 2). FBF was inversely correlated with homeostasis model assessment (HOMA) (*r* = −0.584, *p* < 0.0001), high sensitivity C-reactive protein (hs-CRP) (*r* = −0.528, *p* < 0.0001), waist circumference (*r* = −0.521, *p <* 0.0001), BMI (*r* = −0.505, *p <* 0.0001), PP (*r* = −0.477, *p <* 0.0001), systolic BP (*r* = −0.466, *p* = <0.0001) and age (*r* = −0.319; *p <* 0.0001).

In the MS-NAFLD- group, FBF was significantly correlated with PP (*r* = −0.371, *p <* 0.0001), systolic BP (*r* = −0.361, *p* ≤ 0.0001), HOMA (*r* = −0.362, *p <* 0.0001), hs-CRP (*r* = −0.329, *p <* 0.0001), age (*r* = −0.282; *p* = 0.002), BMI (*r* = −0.279, *p* = 0.002) and waist circumference (*r* = −0.186, *p* = 0.031).

In patients with MS alone, the main covariates related with endothelial-dependent vasodilation were HOMA (*r* = −0.464, *p <* 0.0001), hs-CRP (*r* = −0.446, *p <* 0.0001), waist circumference (*r* = −0.436, *p <* 0.0001), BMI (*r* = −0.406, *p <* 0.0001), PP (*r* = −0.344, *p* = 0.001), age (*r* = −0.305; *p* = 0.003) and systolic BP (*r* = −0.193, *p* = 0.045). Finally, when considering MS and NAFLD together, FBF was inversely correlated with HOMA (*r* = −0.616, *p <* 0.0001), hs-CRP (*r* = −0.522, *p <* 0.0001), waist circumference (*r* = −0.454, *p <* 0.0001), BMI (*r* = −0.414, *p <* 0.0001), PP (*r* = −0.344, *p* = 0.002), systolic BP (*r* = −0.273, *p* = 0.013) age (*r* = −0.249; *p* = 0.022).

Variables reaching statistical significance, with the addition of smoking and gender as dichotomic values, were inserted in a stepwise multivariate linear regression model to determine the independent predictors of FBF (Table 3). In the whole population, HOMA was the strongest predictor of FBF, accounting for 33.7% (*p <* 0.0001) of its variation. In addition, the other independent predictors were: PP, waist circumference, hs-CRP, BMI and age accounting for 8.8%, 5.5%, 3.5%, 1.8%, 1.0% of its variation, respectively.

In subjects without MS and NAFLD, pulse pressure was the most important predictor of FBF, justifying about 12.9% (*p <* 0.0001) of its variation, followed by HOMA (9.9%), hs-CRP (6.8%) and age (4.2%).

Of interest, HOMA was the strongest predictor of FBF in patients with MS alone and MS in combination with NAFLD, accounting for 20.5% (*p <* 0.0001) and 33.2% (*p <* 0.0001) of its variation, respectively. Other independent predictors of the endothelial-dependent vasodilation in MS+NAFLD− group were waist circumference and hs-CRP accounting for a further 8.2% and 6.1% of its variation, respectively. Finally, in the MS+NAFLD+ group, hs-CRP, waist circumference and age add another 11.7%, 7.7% and 3.1% of FBF variation, respectively.

## 3. Discussion

The results of our study, obtained in a well characterized cohort of newly-diagnosed never-treated hypertensive patients, demonstrate that the endothelium-dependent vasodilation, evaluated by strain-gauge plethysmography, was significantly reduced in MS+NAFLD+ patients in comparison with patients with only MS. Furthermore, MS+NAFLD+ patients showed a worse metabolic, inflammatory and hemodynamic profile. In particular, patients with NAFLD exhibited greater values of both BMI and waist circumference compared with those without; this is not surprising, since it is well known that obese subjects have a high risk for NAFLD [18] attributable, at least in part, to visceral fat accumulation and consequent increased flux of free fatty acids to the liver [16]. Moreover, the excessive intrahepatic triglyceride content further impairs insulin sensitivity of these subjects, thus creating a vicious circle explaining the observed metabolic and hemodynamic alterations. This is supported by the finding that, in the linear regression analysis, the main covariate related to FBF was PP in MS−NAFLD− group, while, in the other groups, FBF resulted primarily related to HOMA, regardless of the highest BP values. Moreover, HOMA resulted in being the strongest predictor of FBF both in the MS+NAFLD− and in the MS+NAFLD+ groups, accounting for 20.5% and 33.2% of its variation, respectively. These findings are in agreement with previously published data, confirming the presence of a relationship between impaired endothelium-dependent vasodilation and hypertension [5], as well as a negative effect of MS on vascular function. This is not surprising, since both the hemodynamic and metabolic risk factors configuring the MS are all associated with endothelial dysfunction and, consequently, with the risk of CV events. IR, a condition that can be considered the *leitmotiv* underlying the MS, plays a key role also in the appearance and progression of vascular damage, from the endothelial dysfunction to the atherosclerotic plaque. Moreover, IR is also strongly associated with NAFLD, a condition that can be considered as an epiphenomenon of the interaction between the inflammatory and metabolic factors featuring the MS. Since both endothelial dysfunction and IR are characterized by a reduced endothelial-NO synthase (eNOS)-derived NO bioavailability, it is plausible that the link between NAFLD and endothelial dysfunction could be represented by an altered NO balance. In fact, recent published data [19] demonstrated that NO produced by eNOS, plays a key role in liver physiology and pathophysiology, contributing to the maintenance of liver homeostasis; on the contrary, NO derived from inducible-NO synthase (iNOS) is particularly produced under many pathological conditions, and is able to modify many structural liver proteins. In several pathological conditions, such as IR, NO production is shifted from eNOS- to iNOS-derived, with consequent increase in reactive nitrogen species and free radicals. In particular, Pasarin *et al*. [20] demonstrated that the IR exhibited by a rat model of steatotic liver is particularly expressed at the liver endothelium, thus relating IR to iNOS induction; this IR precedes inflammation, fibrosis or other features of advanced liver disease. In keeping with this, it can be supposed that the impairment of both insulin-induced and ACh-dependent vasodilation seen in peripheral vessels of insulin resistant patients can be also observed in the liver vasculature, thus giving a plausible explanation of many events occurring in the disease progression from NAFLD to cirrhosis [21]. In fact, while insulin acts as a vasodilator agent in physiological conditions, throughout the mediation of NO bioavailability, this property resulted in impaired IR status, due to a combined defect in both insulin-mediated glucose transport and in insulin-stimulated endothelial vasodilation, derived from a fault in the phosphatidylinositol 3 kinase/Akt pathway. [22]. Moreover, the findings of the present study strengthen previously published data by our group [17], demonstrating a significant reduction in endothelium-dependent vasodilation evaluated by strain-gauge plethysmography in hypertensives with associated NAFLD, compared with hypertensives without NAFLD. All these data, taken together, endorse the close link between IR and NAFLD observed in other pathological conditions such as type-2 diabetes mellitus, obesity, and other metabolic alterations [14,23,24]. Finally, our data, obtained in a well-characterized population of hypertensive patients, are in agreement with those obtained by Targher and co-workers in diabetic patients, demonstrating that non-alcoholic fatty liver disease significantly increases CV risk in this setting of patients [25].

This study has several potential limitations. First of all, the small sample size and the cross-sectional design impose the data obtained to be confirmed in wider trials. Another limitation is that the diagnosis of NAFLD was performed by using the non-invasive fatty liver index (FLI) instead of liver biopsy that represents the gold standard. In fact, FLI is poorly correlated with liver histology [26], is no better than waist circumference in predicting NAFLD [27], and the pathophysiological information from the NAFLD arena cannot be directly extrapolated and applied to “liver steatosis” of undefined etiology (probably a mixture of alcoholic and nonalcoholic fatty liver disease), although some authors believe that steatosis *per se* may enhance CV risk [28]. Finally, in this study, we determined IR by using the HOMA index that does not allow for discrimination between peripheral or central IR.

In conclusion, we demonstrated that hypertensive patients with both MS and NAFLD show a worst endothelium-dependent vasodilation compared with hypertensives with MS alone, thus enhancing the crucial role of IR in the multifactorial pathway, in which cooperate both metabolic and hemodynamic factors, leading from endothelial dysfunction to the atherosclerotic plaque formation.

Thus, our results have an important clinical implication since allow to consider NAFLD not only as an organ damage consequent to IR, but also a simple and early marker of endothelial dysfunction in essential hypertension, contributing to better stratify CV risk in this setting of patients. 

## 4. Materials and Methods

### 4.1. Study Population

The study population consisted of outpatients evaluated at the University Hospital of Catanzaro. We recruited 272 Caucasian newly-diagnosed never-treated hypertensive outpatients (148 males and 124 females) divided into three groups according with the presence or absence of MS alone or in combination with NAFLD (MS−NAFLD−, MS+NAFLD−, MS+NAFLD+). All patients participated in the CATAnzaro MEtabolic RIsk Factors Study (CATAMERIS) [29] and underwent physical examination and review of their medical history. None of the patients had history or clinical evidence of chronic hepatitis, alcoholism, coronary artery disease, valvular heart disease, peripheral vascular disease, coagulopathy, or any disease predisposing to vasculitis or Raynaud’s phenomenon. A complete anthropometric assessment was performed by measurements of height, weight, and waist circumference according to a standardized protocol. BMI was calculated as kilograms per square meter, and the waist was measured at its smallest point with the abdomen relaxed.

The MS was defined according to NCEP-ATPIII [1]. The presence of NAFLD was detected calculating the non-invasive FLI, as suggested by Bedogni *et al*. [30], according to the formula:

FLI = (e ^0.953*loge (triglyceride) + 0.139*BMI + 0.718*loge (GGT) + 0.053*waist circumference − 15.745^)/(1 + e ^0.953*loge (triglyceride) + 0.139*BMI + 0.718*loge (GGT) + 0.053*waist circumference − 15.745^) * 100.

FLI values ≥60 are significant to rule in fatty liver as detected by ultrasonography. The protocol was approved by the Local Ethical Committee, and all participants gave their informed written consent before the study procedures. All the investigations of this research protocol were performed in accordance with the principles of the Declaration of Helsinki.

### 4.2. Biochemical Assays

All laboratory determinations were obtained after 12 fasting h. Enzymatic methods were used to measure fasting blood glucose, total and HDL-cholesterol, and triglyceride (Roche Diagnostics, Mannheim, Germany). ALT and AST levels were measured using the α-ketoglutarate reaction; GGT levels with the l-γ-glutamyl-3-carboxy-4-nitroaniliderate method. Serum insulin was measured through a highly specific radioimmunoassay using two monoclonal antibodies; intra-assay coefficient of variation (CofV) 2.1%, inter-assay CofV 2.9%. hs-CRP was measured by a high-sensitivity turbidimetric immunoassay (Behring, Marburg, Germany). Creatinine measurements were performed by use of the Jaffe methodology and the uricase/peroxidase (uricase/POD; Boehringer Mannheim, Mannheim, Germany) method implemented in an auto-analyzer. Renal function was evaluated by estimated glomerular filtration rate (e-GFR) by using the Chronic Kidney Disease – Epidemiology (CKD-EPI) equation [31]. Insulin sensitivity was estimated by using the HOMA index, calculated according to the formula: HOMA = [insulin (μU/mL) × glucose (mmol/L)]/22.5. The HOMA index has a strict correlation with the measurement of insulin sensitivity obtained directly from the euglycemic clamp [32,33].

### 4.3. Blood Pressure Measurements

Clinical BP readings were obtained with a mercury sphygmomanometer in the left arm of patients lying supine, after 5 minutes of quiet rest. Each patient underwent a minimum of three BP measurements on three separate occasions at least two weeks apart. The average of the last two of three consecutive measurements obtained at intervals of three minutes was considered as baseline BP. Systolic and diastolic BP corresponded with the first appearance (phase I) and the disappearance (phase V) of Korotkfoff sounds, respectively. According to current guidelines, patients with a clinical BP ≥ 140 mmHg systolic and/or 90 mmHg diastolic were defined as hypertensive [34].

### 4.4. Forearm Blood Flow Measurements

All studies were performed at 09:00 A.M. after overnight fasting, with the subjects lying supine in a quiet air-conditioned room (22–24 °C). Subjects continued their regular diet, but were advised to stop caffeine, alcohol and smoking at least 24 h before the study. Forearm volume was determined by water displacement. A 20-gauge polyethylene catheter (Vasculon 2) was inserted, under local anesthesia and sterile conditions, into the brachial artery of the non-dominant arm for both BP evaluation (Baxter Healthcare Corp., Deerfield, IL, USA) and drug infusion. This arm was elevated above the level of the right atrium, and a mercury-filled elastic strain-gauge, connected to a plethysmograph (model EC-4, D.E. Hokanson, Issaquah, WA, USA) calibrated to measure the percent change in volume which was, in turn, connected to a chart recorder to obtain FBF measurements, was placed on the widest part of the forearm. To exclude venous outflow, a cuff placed on the upper arm was inflated to 40 mmHg with a rapid cuff inflator (model E-10, Hokanson, Issaquah, WA, USA). The hand blood flow was excluded by inflating a wrist cuff to BP values 1 min before each measurement. The antecubital vein in the opposite arm was cannulated. The FBF was measured as the slope of the change in the forearm volume [35]. The mean of at least three measurements was obtained at each time point.

### 4.5. Vascular Function

For the present study, we used the protocol previously described by Panza *et al*. [36], and subsequently used by our group [5,6,7,8,9,11,12,37]. For each patient, we obtained measurements of FBF and BP during intra-arterial infusion of saline, ACh and SNP at increasing doses. ACh (Sigma, Milan, Italy) was diluted with saline immediately before infusion. SNP (Malesci, Florence, Italy) was diluted in 5% glucose solution immediately before each infusion and protected from light with aluminium foil. To reach a stable baseline before data collection, all participants rested for 30 min after artery cannulation; measurements of FBF were repeated every 5 min until stable. We assessed endothelium-dependent and endothelium-independent vasodilation by a dose–response curve to intra-arterial ACh infusions (7.5, 15, and 30 µg/mL per min, each for 5 min) and SNP infusions (0.8, 1.6, and 3.2 µg/mL per min, each for 5 min), respectively. To avoid any bias related to drug infusion, the sequence of administration of ACh and SNP was randomized. The drug infusion rate, adjusted for the forearm volume of each subject, was 1 mL/min.

### 4.6. Statistical Analysis

Differences for clinical and biological data were compared by using analysis of variance (ANOVA), Bonferroni *post hoc t*-test and chi-square test, as appropriate. The vasodilating responses to ACh and SNP were compared by one-way ANOVA and, when analysis was significant, the Bonferroni *post hoc t*-test was applied. A logistic regression analysis was performed to test the risk for decreased FBF (defined by values <300 mL·100 mL^−1^ of tissue·min^−1^) in presence of NAFLD and MS.

Linear regression analysis was performed to correlate FBF with the following covariates: age, waist circumference, BMI, systolic BP, diastolic BP, PP, total and LDL- and HDL-cholesterol, triglyceride, hs-CRP, HOMA. To define the independent predictors of FBF, variables reaching statistical significance were inserted in a stepwise multivariate linear regression model. Moreover, to avoid a possible colinearity, we considered only HOMA and not fasting glucose and insulin.

Parametric data are reported as mean ± SD. Significant differences were assumed to be at *p <* 0.05. All comparisons were performed using the statistical package SPSS 21.0 for Mac (Manufacturer, City, Country).

## Figures and Tables

**Table 1:** Clinical, biochemical and hemodynamic characteristics of subjects in whole study population and in different groups.

Variables	All (*n* = 272)	MS−NAFLD− (*n* = 101)	MS+NAFLD− (*n* = 78)	MS+NAFLD+ (*n* = 93)	*p*
Gender, M/F	148/124	63/38	37/41	48/45	0.110 *
Age, years	48.8 ± 9.3	47.2 ± 8.6	49.9 ± 10.3	49.9 ± 9.1	0.082
Smoking, *n* (%)	17 (17.3)	17 (16.8)	14 (17.9)	16 (17.2)	0.960 *
BMI, kg/m^2^	30.1 ± 5.4	26.2 ± 2.5	31.2 ± 4.8	33.3 ± 5.6 ^‡^	<0.0001
Waist circumference, cm	100.5 ± 14.2	90.2 ± 10.8	104.1 ± 13.2	108.5 ± 11.6 ^‡^	<0.0001
Systolic BP, mm Hg	141 ± 17	129 ± 13	145 ± 17	150 ± 14 ^‡^	<0.0001
Diastolic BP, mm Hg	89 ± 11	83 ± 10	92 ± 12	92 ± 10	<0.0001
PP, mm/Hg	52 ± 14	46 ± 15	52 ± 13	59 ± 14 ^‡^	<0.0001
Total cholesterol, mg/dL	197 ± 33	186 ± 26	204 ± 32	205 ± 37	<0.0001
HDL-cholesterol, mg/dL	47 ± 14	53 ± 16	43 ± 10	43 ± 11	<0.0001
Triglyceride, mg/dL	132 ± 63	107 ± 42	134 ± 65	156 ± 81	<0.0001
GGT, U/L	31 ± 15	21 ± 7	26 ± 8	47 ± 11 ^‡^	<0.0001
AST, U/L	37.7 ± 24.1	19.4 ± 4.6	30.8 ± 18.3	63.9 ± 20.9 ^‡^	<0.0001
ALT, U/L	39.4 ± 27.4	18.8 ± 6.2	31.2 ± 16.5	69.2 ± 22.7 ^‡^	<0.0001
Serum Creatinine, mg/dL	0.9 ± 0.2	0.9 ± 0.3	0.9 ± 0.2	0.9 ± 0.2	0.162
e-GFR, mL/min/1.73 m^2^	94.7 ± 20.6	97.1 ± 21.3	93.1 ± 18.7	92.9 ± 25.6	0.287
FP glucose, mg/dL	99.5 ± 19.6	90.1 ± 8.3	102.2 ± 21.9	107.9 ± 21.9	<0.0001
FP insulin, mU/mL	13.7 ± 6.3	10.3 ± 4.7	14.5 ± 6.0	16.8 ± 6.4 ^‡^	<0.0001
HOMA	3.4 ± 1.9	2.3 ± 1.0	3.6 ± 1.5	4.5 ± 2.2 ^‡^	<0.0001
hs-CRP, mg/dL	4.3 ± 2.7	3.2 ± 1.5	4.2 ± 3.0	5.7 ± 2.9 ^‡^	<0.0001
FBF, mL·100·mL^−1^ of tissue·min^−1^					
Basal	3.1 ± 0.7	3.2 ± 0.9	3.0 ± 0.6	3.0 ± 0.7	0.238
ACh, % of increase	328 ± 141	413 ± 136	327 ± 127	236 ± 91 ^‡^	<0.0001
SNP, % of increase	500 ± 120	507 ± 128	498 ± 121	496 ± 114	0.799

* : Χ^2^ test. ^‡^ : = *p* < 0.05 by Bonferroni MS+NAFLD− Vs MS+NAFLD+. ACh: acetylcholine; ALT alanine aminotransferase; AST: aspartate aminotransferase; BMI: body mass index; BP: blood pressure; PP: pulse pressure; hs-CRP: high sensitivity C-reactive protein; e-GFR: estimated glomerular filtration rate; FBF: forearm blood flow; FP: fasting plasma; GGT: gamma glutamyl transferase; HDL: high density lipoprotein; HOMA: homeostasis model assessment of insulin resistance; SNP: sodium nitroprusside.

**Table 2:** Linear regression analysis on forearm blood flow (FBF) as a dependent variable in the whole study population and in different groups.

Variables	All		MS−NAFLD−		MS+NAFLD−		MS+NAFLD+	
	*n =* 272		*n =* 101		*n =* 98		*n =* 73	
	*r*	*p*	*r*	*p*	*r*	*p*	*r*	*p*
Diastolic BP, mmHg	−0.138	ns	0.053	ns	0.122	ns	0.050	ns
HDL cholesterol, mg/dL	0.171	ns	0.125	ns	−0.171	ns	0.001	ns
Total cholesterol, mg/dL	−0.171	ns	−0.094	ns	0.016	ns	0.007	ns
Triglyceride, mg/dL	−0.202	ns	−0.114	ns	−0.005	ns	0.068	ns
Age, years	−0.319	<0.0001	−0.282	0.002	−0.305	0.003	−0.249	0.022
Systolic BP, mmHg	−0.466	<0.0001	−0.361	<0.0001	−0.193	0.045	−0.273	0.013
PP, mmHg	−0.477	<0.0001	−0.371	<0.0001	−0.344	0.001	−0.344	0.002
BMI, kg/m^2^	−0.505	<0.0001	−0.279	0.002	−0.406	<0.0001	−0.414	<0.0001
Waist circumference, cm	−0.521	<0.0001	−0.186	0.031	−0.436	<0.0001	−0.454	<0.0001
hs-CRP, mg/dL	−0.528	<0.0001	−0.329	<0.0001	−0.446	<0.0001	−0.522	<0.0001
HOMA	−0.584	<0.0001	−0.362	<0.0001	−0.464	<0.0001	−0.616	<0.0001

BP: blood pressure; PP: pulse pressure; HDL: high-density lipoprotein; BMI: body mass index; hs-CRP: high-sensitivity C-reactive protein; HOMA: homeostasis model assessment of insulin resistance.

**Table 3:** Stepwise multiple regression analysis FBF as a dependent variable in the whole study population and in different groups.

Variables	All (*n* = 272)		MS–NAFLD– (*n* = 101)		MS+NAFLD– (*n* = 98)		MS+NAFLD+ (*n* = 73)	
	Partial *R*^2^ (%)	*p*	Partial *R*^2^ (%)	*p*	Partial *R*^2^ (%)	*p*	Partial *R*^2^ (%)	*p*
Age, years	1.0	0.012	4.2	0.008	-	-	3.1	0.021
BMI, kg/m^2^	1.8	0.001	-	-	-	-	-	-
hs-CRP, mg/dL	3.5	<0.0001	6.8	0.002	6.1	0.006	11.7	<0.0001
Waist circumference, cm	5.5	<0.0001	-	-	8.2	0.003	7.7	0.001
PP, mmHg	8.8	<0.0001	12.9	<0.0001	-	-	-	-
HOMA	33.7	<0.0001	9.9	<0.0001	20.5	<0.0001	33.2	<0.0001
Total *R*^2^ (%)	54.3	-	33.8	-	34.8	-	55.7	-

BMI: body mass index; hs-CRP: high-sensitivity C-reactive protein; PP: pulse pressure; HOMA: homeostasis model assessment of insulin resistance.

## Abbreviations

MS	metabolic syndrome
CV	cardiovascular
IR	insulin resistance
NO	nitric oxide
NAFLD	non-alcoholic fatty liver disease
BMI	body mass index
PP	pulse pressure
BP	blood pressure
GGT	gamma-glutamyltransferase
AST	gamma-glutamyltransferase
ALT	alanine aminotransferase
FBF	forearm blood flow
ACh	achetylcholine
SNP	sodium nitroprusside
HOMA	homeostasis model assessment
hs-CRP	high sensitivity C-reactive protein
eNOS	endothelial-nitric oxide synthase
iNOS	inducible-nitric oxide synthase
FLI	fatty liver index
eGFR	estimated glomerular filtration rate
